# Evaluating the Health-Related Quality of Life in Patients with COPD and Chronic Heart Failure Post-Hospitalization after COVID-19 Using the EQ-5D and KCCQ Questionnaires

**DOI:** 10.3390/diseases12060124

**Published:** 2024-06-07

**Authors:** Ilona Emoke Sukosd, Sai Teja Gadde, Myneni Pravallika, Silvius Alexandru Pescariu, Mihaela Prodan, Ana-Olivia Toma, Roxana Manuela Fericean, Ingrid Hrubaru, Oana Silvana Sarau, Ovidiu Fira-Mladinescu

**Affiliations:** 1 Doctoral School, Department of General Medicine, Victor Babeş University of Medicine and Pharmacy, 300041 Timisoara, Romania; sukosd.emoke@umft.ro (I.E.S.); oana.sarau@umft.ro (O.S.S.); 2 Center for Research and Innovation in Precision Medicine of Respiratory Diseases, Victor Babeş University of Medicine and Pharmacy, Eftimie Murgu Square 2, 300041 Timisoara, Romania; mihaela.prodan@umft.ro (M.P.); mladinescu@umft.ro (O.F.-M.); 3 Faculty of General Medicine, All India Institute of Medical Sciences (AIIMS), Mangalagiri 522503, India; gaddesai0626@gmail.com; 4 Katuri Medical College and Hospital, Chinakondrupadu 522019, India; myneni2737@gmail.com; 5 Department of Cardiology, Victor Babeş University of Medicine and Pharmacy, 300041 Timisoara, Romania; pescariu.alexandru@umft.ro; 6 Department of Plastic Surgery, “Pius Brinzeu” Timis County Emergency Clinical Hospital, 300723 Timisoara, Romania; 7 Discipline of Dermatology, Victor Babeş University of Medicine and Pharmacy, 300041 Timisoara, Romania; 8 Department of Dermatology, Timisoara Municipal Emergency Hospital, 300254 Timisoara, Romania; manuela.fericean@umft.ro; 9 Department of Obstetrics and Gynecology, Victor Babeş University of Medicine and Pharmacy, 300041 Timisoara, Romania; hrubaru.ingrid@umft.ro; 10 Department V, Internal Medicine, Discipline of Hematology, Victor Babeş University of Medicine and Pharmacy, Eftimie Murgu Square 2, 300041 Timisoara, Romania; 11 Department of Infectious Diseases, Discipline of Pulmonology, Victor Babeş University of Medicine and Pharmacy, Eftimie Murgu Square 2, 300041 Timisoara, Romania

**Keywords:** heart failure, chronic obstructive pulmonary disease, quality of life

## Abstract

Chronic heart failure (CHF) and chronic obstructive pulmonary disease (COPD) frequently coexist, significantly impacting health-related quality of life (HRQoL). This study evaluated HRQoL in patients with CHF, COPD, or both, three months post-COVID-19 discharge using EQ-5D and KCCQ questionnaires to guide targeted healthcare interventions. We conducted a cross-sectional study at “Victor Babes” Hospital in Timisoara, enrolling 180 patients who had recovered from COVID-19 (60 in each group including CHF, COPD, and both conditions). HRQoL was assessed via EQ-5D and KCCQ. Significant disparities in HRQoL measures were noted across the groups. Patients with both CHF and COPD reported the worst outcomes, especially in terms of hospital stay lengths due to COVID-19 (11.63 days) and initial oxygen saturation levels (88.7%). HRQoL improvements from discharge to three months post-discharge were significant, with EQ-5D mobility scores improving notably across all groups (CHF and COPD: 2.87 to 2.34, *p* = 0.010). KCCQ results reflected substantial enhancements in physical limitation (CHF and COPD: 38.94 to 58.54, *p* = 0.001) and quality of life scores (CHF and COPD: 41.38 to 61.92, *p* = 0.0031). Regression analysis revealed that dual diagnosis (CHF and COPD) significantly impacted usual activities and quality of life (β = −0.252, *p* = 0.048; β = −0.448, *p* = 0.017), whereas the initial severity of COVID-19 was a significant predictor of worse HRQoL outcomes (β = −0.298, *p* = 0.037; β = −0.342, *p* = 0.024). The presence of both CHF and COPD in patients recovering from COVID-19 was associated with more severe HRQoL impairment compared with either condition alone. These findings emphasize the need for specialized, comprehensive post-COVID-19 recovery programs that address the complex interplay among chronic conditions to optimize patient outcomes and enhance quality of life.

## 1. Introduction

Chronic heart failure (CHF) and chronic obstructive pulmonary disease (COPD) are two prevalent conditions that significantly impact global health [1,2]. Both diseases are associated with high morbidity and mortality rates and lead to substantial degradation in the quality of life of affected individuals [3,4,5]. Despite their distinct pathophysiological bases, there is a high incidence of comorbidity between CHF and COPD, which complicates their management and affects patient outcomes [6]. Moreover, the COVID-19 pandemic showed that patients with chronic diseases were more likely to experience severe complications of SARS-CoV-2 infection and post-COVID-19 syndrome [7,8].

Quality of life (QoL) is a critical outcome in the management of chronic diseases, offering insight into the effectiveness of medical interventions and the overall burden of disease on daily functioning and well-being [9,10]. The EQ-5D questionnaire, a standardized instrument developed by the EuroQol Group, is widely used to measure health-related quality of life (HRQoL) and provides a comprehensive method for assessing general health status [11]. By focusing on five key dimensions of health, the EQ-5D facilitates a detailed understanding of how diseases like CHF and COPD affect patients beyond traditional clinical outcomes [12,13].

COPD itself is a major cause of disability and a critical factor in the health deterioration of individuals, particularly when co-existing with other chronic conditions such as heart failure [14,15]. The presence of COPD in CHF patients can lead to worse health outcomes, increased hospitalization rates, and a more complicated disease management scenario [16]. Understanding how COPD exacerbates heart failure symptoms and impacts the progression of CHF is crucial for developing tailored treatment strategies that address both conditions simultaneously.

Considering patients with chronic diseases, the assessment of HRQoL is paramount in understanding the full impact of illness on patient well-being. Several prominent tools are used in clinical and research settings to measure HRQoL, including the Short Form Health Survey (SF-36) [17], the Health Utilities Index (HUI) [18], and the Quality of Well-Being Scale (QWB) [19]. Each tool has its unique focus and method of capturing patient-reported health outcomes. Among these, the EQ-5D stands out because of its simplicity, comprehensiveness, and adaptability across diverse patient groups and health conditions [20]. The EQ-5D questionnaire evaluates the following five dimensions of health: mobility, self-care, usual activities, pain/discomfort, and anxiety/depression, which collectively offer a holistic view of a patient’s health status. Similarly, the KCCQ questionnaire proved to be an important and useful tool for evaluating quality of life in chronic heart disease.

The current study aims to investigate and compare the quality of life among three distinct groups of patients with different comorbid conditions, those with CHF, those with COPD, and those with both conditions, three months after SARS-CoV-2 infection. The objective is to determine the differential impact of these chronic conditions on recovery after acute COVID-19, which will provide insights for targeted healthcare interventions to enhance recovery and quality of life in these populations.

## 2. Materials and Methods

### 2.1. Research Design

This study implemented a cross-sectional research design to examine the impact of COVID-19 on the disease outcomes and health-related quality of life in patients with COPD and CHF, assessed using the EQ-5D and KCCQ questionnaires. This approach was chosen to trace the progression of both conditions and their combined effects on patient health status after SARS-CoV-2 infection, assessed three months post-hospital discharge. Participants were recruited from the Cardiology and Pulmonology Departments of the “Victor Babes” Hospital in Timisoara, as well as the Institute of Cardiovascular and Heart Diseases of Timisoara, affiliated with the “Victor Babes” University of Medicine and Pharmacy Timisoara.

All patients included in this study were diagnosed with COVID-19 after positive screening for the viral infection through RT-PCR. The main study group consisted of patients with CHF (NYHA I and II), according to the NYHA guidelines [21], and had a documented history of COPD GOLD A and B according to the Global Initiative for Chronic Obstructive Lung Disease [22]. The first control group consisted of patients with CHF-only (NYHA I and II), while the second control group comprised patients with COPD-only GOLD A and B. The selection of NYHA I and II and GOLD A and B patients was considered to avoid the confounding effect of disease severity on health-related quality of life. The recruitment process began following a detailed review of their medical records to confirm eligibility based on predefined inclusion and exclusion criteria. The criteria ensured that this study would be representative of the target population, enhancing the generalizability of the findings.

Ethical approval for this research was obtained from the Institutional Review Board of the Central University of Medical Sciences, which adheres to the ethical standards set forth in the 1964 Declaration of Helsinki and its later amendments, the EU GCP Directive 2001/20/EC, and ICH Good Clinical Practice guidelines. Prior to participation, all patients provided written informed consent, which detailed this study’s objectives, the nature of the data to be collected, their right to withdraw at any time without penalty, and measures taken to ensure confidentiality and data protection. This consent process was conducted in accordance with the ethical principles of autonomy and respect for persons, ensuring that all participants were fully informed and voluntarily participating.

### 2.2. Inclusion Criteria

The inclusion criteria for this study were designed to select a homogeneous group of participants who could provide clear insights into the impact of COPD on chronic heart failure outcomes. Eligible participants were adults aged 40 years and above, diagnosed with CHF according to the ESC guidelines [23]. Additionally, all participants were required to have a confirmed diagnosis of COPD, as defined by the GOLD standards, including documented spirometry results. Participants needed to be under active management for both conditions at the time of recruitment and capable of giving informed consent. They also had to have a stable health status without hospital admission for acute exacerbations of either CHF or COPD in the three months preceding study enrollment.

Exclusion criteria were established to eliminate potential confounders that could impact the understanding of the interrelationship between COPD and CHF. Patients were excluded if they had significant comorbid conditions such as active malignancy, end-stage renal disease requiring dialysis, or severe cognitive impairment that could interfere with the ability to provide informed consent or adhere to study protocols. Individuals with a life expectancy of less than one year, as determined by their primary care physician, were also excluded. Additionally, patients who were participating in other clinical trials or studies that could influence outcomes related to CHF or COPD management were not eligible for inclusion. Other exclusion criteria were considered for patients from NYHA III and IV CHF disease severity and GOLD C and D COPD disease severity. Patients who were currently smoking were also excluded from this study, as well as those who did not have a confirmed diagnosis of COVID-19 assessed with RT-PCR 3 months before completing the questionnaires.

### 2.3. Variables

In this study, a comprehensive range of variables was collected to analyze the impact of COPD on disease outcomes and health-related quality of life in patients with chronic heart failure, assessed using the EQ-5D and KCCQ questionnaires. Demographic data included sex and age. Clinical assessments encompassed heart failure classification (NYHA class), comorbidities, presence of COPD/asthma prior to this study, and diabetes, along with symptoms such as dyspnea, edema, and chest pain. Physiological measurements recorded were oxygen saturation, systolic and diastolic blood pressure, body temperature, and heart rate. Additional clinical parameters included length of hospital stay, pulmonary involvement from CT scans, ECG, ejection fraction (EF), and laboratory tests such as brain natriuretic peptide, D-dimers, hemoglobin, white blood cell count, lymphocytes, high-sensitivity C-reactive protein, creatinine, and blood glucose. Medication usage data were also collected, including furosemide, beta-blockers, ACE inhibitors, and spironolactone.

### 2.4. Study Questionnaires

The EQ-5D is a standardized instrument used internationally for measuring generic health status. It comprises five dimensions including mobility, self-care, usual activities, pain/discomfort, and anxiety/depression. Each dimension has three levels including no problems, some problems, and extreme problems. The responses provide a simple descriptive profile and a single index value for health status, which can be used in clinical and economic appraisal. The KCCQ, specifically designed for patients with heart failure, assesses specific health domains that are critical to these patients. It measures physical limitations, symptoms (frequency, severity, and recent change), self-efficacy, social interference, and quality of life. Scores range from 0 to 100, with higher scores indicating better health status.

### 2.5. Statistical Analysis

Data management and statistical analyses for this study were performed using the statistical software R version 4.1 (R Foundation for Statistical Computing, Vienna, Austria). The sample size was determined using a convenience sampling method, aiming for a minimum of 67 patients to achieve a 90% confidence level with a 10% margin of error. Continuous variables following a normal distribution were presented as mean ± standard deviation (SD), whereas those not normally distributed were described using median and interquartile range (IQR). Categorical variables were summarized as frequencies and percentages. For comparing the means of more than two groups of normally distributed continuous variables, ANOVA was applied, and for non-normally distributed data, the Kruskal–Wallis test was used. The Chi-square test was utilized to analyze categorical variables. Multiple regression analysis was conducted to investigate the impacts of COPD on the outcomes in CHF patients. A *p*-value of less than 0.05 was considered statistically significant. All data analyses were rigorously verified to ensure the accuracy and reliability of the results.

## 3. Results

### 3.1. Background Analysis

A total of 180 patients were included in the final analysis, after including the completed surveys and matching the cases. In the analysis of patient demographics and health characteristics across three groups—patients with both chronic heart failure (CHF) and chronic obstructive pulmonary disease (COPD), patients with only CHF, and patients with only COPD—no significant differences were noted in age or gender distributions. Specifically, the average ages were 63.81, 65.22, and 66.96 years, respectively, with no statistically significant variance (*p* = 0.221). Similarly, the proportions of females in each group were 43.33%, 56.67%, and 46.67%, respectively, showing no significant differences (*p* = 0.4225). Significant disparities were observed in smoking histories and associated pack-years, with 86.67% of the combined CHF and COPD group, 13.33% of the CHF-only group, and 90% of the COPD-only group having a history of smoking (*p* = 0.0001). The mean pack-year values were 22.83, 2.08, and 30.86, respectively, also showing significant differences (*p* = 0.0003).

Among specific comorbidities, notable differences were seen in the prevalence of asthma and ischemic heart disease, with asthma being notably more prevalent in the COPD group (66.67%) compared with the CHF and combined groups (16.67% and 36.67%, respectively; *p* = 0.0057). Ischemic heart disease was most common in the CHF group at 58.33%, compared with 43.33% and 25.00% in the combined and COPD groups, respectively (*p* = 0.0005). The analysis further showed significant differences in days hospitalized due to COVID-19, with the combined group experiencing a longer mean duration (11.63 days) compared with the CHF (8.04 days) and COPD groups (9.97 days) (*p* = 0.0001). Other variables, including NYHA and GOLD classifications, diabetes mellitus, and other comorbidities did not show significant differences or presented mixed results (Table 1).

### 3.2. Disease Assessment

Oxygen saturation levels differed significantly, with the CHF and COPD groups registering the lowest mean saturation at 88.7%, compared with 92.4% in the CHF group and 89.9% in the COPD group (*p* = 0.0075). The temperature at admission also showed significant variance, with the CHF and COPD group averaging higher at 38.4 °C, compared with 37.9 °C in the CHF group and 38.2 °C in the COPD group (*p* = 0.034). The extent of lung involvement on CT scans and ejection fraction (EF) measurements revealed notable discrepancies. Lung involvement was significantly greater in the CHF and COPD groups, averaging 56.2%, compared with 42.2% in the CHF group and 51.5% in the COPD group (*p* = 0.0002). EF was markedly lower in the combined CHF and COPD group at 38.4%, versus 40.6% in the CHF group and 53.2% in the COPD group (*p* = 0.0002). Additionally, the prevalence of abnormal electrocardiograms (ECGs) was substantially higher in the combined group at 81.67%, contrasted with 38.33% in the CHF group and 56.67% in the COPD group (*p* < 0.001), as presented in Table 2.

At three months post-admission, oxygen saturation levels varied significantly, with the CHF and COPD groups recording the lowest average saturation at 92.6%, compared with 94.6% in the CHF group and 92.4% in the COPD group (*p* < 0.001). Diastolic blood pressure also showed significant differences, averaging 83.0 mmHg in the CHF and COPD group, 79.5 mmHg in the CHF group, and 81.0 mmHg in the COPD group (*p* = 0.001). Body temperature differences were notable, with the CHF and COPD group averaging 37.3 °C, compared with 36.7 °C in the CHF group and 37.4 °C in the COPD group (*p* < 0.001). Lung involvement on CT scans indicated significant differences, with the CHF and COPD group showing more extensive involvement at 22.5%, compared with 16.0% in the CHF group and 20.5% in the COPD group (*p* < 0.001). The ejection fraction (EF) also showed marked differences, with the CHF and COPD group at 40.6%, the CHF group at 42.3%, and the COPD group at 54.1% (*p* < 0.001).

Furthermore, the prevalence of abnormal electrocardiograms (ECGs) was significantly higher in the CHF and COPD group at 73.33%, compared with 30.00% in the CHF group and 56.67% in the COPD group (*p* < 0.001). Current signs and symptoms like dyspnea and tiredness were notably more prevalent in the CHF and COPD group, with dyspnea affecting 71.67% and tiredness 76.67%, significantly higher than in the other groups (*p* = 0.003 for dyspnea; *p* < 0.001 for tiredness), as presented in Table 3.

### 3.3. Analysis of Questionnaires

Mobility scores improved significantly over time across all groups. The CHF and COPD group’s scores decreased from 2.87 to 2.34 (*p* = 0.010 for the Time*Group interaction), CHF from 2.14 to 1.67, and COPD from 2.63 to 2.08. The results indicate significant group differences (*p* = 0.004) and an overall improvement over time (*p* < 0.001). Self-care also showed marked improvements. The scores for the CHF and COPD group improved from 2.57 to 2.03 (*p* = 0.033 for the Time*Group interaction). For CHF, the scores decreased from 1.92 to 1.54, and for COPD, from 2.29 to 1.87. Significant group differences (*p* = 0.020) and overall time improvement (*p* < 0.001) were observed.

Usual activities experienced noticeable improvements as well. The CHF and COPD group’s scores decreased from 3.03 to 2.46 (*p* = 0.008 for the Time*Group interaction). CHF scores dropped from 2.18 to 1.73, and COPD from 2.72 to 2.19. This domain showed significant variations among the groups (*p* = 0.001) and over time (*p* < 0.001). Pain/discomfort levels also decreased, with the CHF and COPD group moving from 2.68 to 2.17 (*p* = 0.027 for the Time*Group interaction). CHF scores improved from 2.07 to 1.61, and COPD from 2.36 to 1.94 (*p* < 0.001). Anxiety/depression scores saw notable improvements as well. The CHF and COPD group’s scores reduced from 2.98 to 2.47 (*p* = 0.009 for the Time*Group interaction). CHF scores decreased from 1.84 to 1.38, and COPD from 2.41 to 1.96 (*p* < 0.001), as presented in Table 4.

Regarding physical limitations, the CHF and COPD group improved from 38.94 to 58.54 (*p* = 0.0010 for the Time*Group interaction), CHF from 53.84 to 74.02, and COPD from 48.38 to 68.27. Significant differences were found among the groups (*p* = 0.006) with overall improvement over time (*p* < 0.001). There were significant enhancements in symptom scores. The CHF and COPD group’s scores rose from 43.47 to 63.73 (*p* = 0.0026 for the Time*Group interaction), CHF from 58.91 to 78.36, and COPD from 53.07 to 71.52. This dimension showed significant group differences (*p* = 0.011) and an overall time improvement (*p* < 0.001). The CHF and COPD group saw an increase from 41.38 to 61.92 (*p* = 0.0031 for the Time*Group interaction), CHF from 56.62 to 76.38, and COPD from 50.67 to 70.14. Both group differences (*p* = 0.009) and the time effect (*p* < 0.001) were significant.

In social limitation, all groups showed improvements. The CHF and COPD group’s scores rose from 31.97 to 51.86 (*p* = 0.001 for the Time*Group interaction), CHF from 46.48 to 66.69, and COPD from 42.88 to 62.39. Significant group effects (*p* = 0.001) and a strong main effect for time (*p* < 0.001) were noted. In terms of self-efficacy, significant increases were also seen in self-efficacy scores. The CHF and COPD group improved from 46.28 to 67.09 (*p* = 0.001 for the Time*Group interaction), CHF from 60.96 to 81.76, and COPD from 55.62 to 73.24. There were notable group differences (*p* = 0.009) and a significant improvement over time (*p* < 0.001) (Table 5).

Patients with both CHF and COPD showed statistically significant negative impacts on both usual activities (β = −0.252, *p* = 0.048) and quality of life (β = −0.448, *p* = 0.017). In contrast, neither CHF nor COPD alone had a significant effect on usual activities, and the negative influence of COPD on quality of life approached significance but did not reach the conventional threshold (*p* = 0.083). The initial severity of COVID-19 significantly influenced outcomes, correlating with worse results in both performing usual activities (β = −0.298, *p* = 0.037) and maintaining quality of life (β = −0.342, *p* = 0.024). These findings underscore the lasting impact that initial health status during acute illness can have on recovery and overall quality of life (Table 6).

## 4. Discussion

### 4.1. Literature Findings

The findings of our study illustrate a pronounced impact of combined chronic heart failure and chronic obstructive pulmonary disease on various health metrics three months post-COVID-19. Notably, oxygen saturation levels among the CHF and COPD group were significantly lower compared with the CHF-only group, underscoring the exacerbated respiratory compromise in patients managing both conditions. This dual diagnosis group also showed a higher mean body temperature, which could indicate a prolonged inflammatory or infection response, a common complication in patients with more severe cardiopulmonary disease.

Moreover, the lung involvement observed through CT scans was significantly more pronounced in the CHF and COPD group than in either condition alone. This finding aligns with known pathophysiological interactions where CHF can exacerbate the pulmonary consequences of COPD, and vice versa, leading to more severe symptoms and complications. This severe lung involvement could be a critical factor in the lower oxygen saturation levels observed, illustrating how compounded chronic conditions can lead to a more challenging recovery from acute events like COVID-19.

Significantly lower ejection fractions in the CHF and COPD group compared with the CHF-only and COPD-only groups highlight the severe cardiac impairment in patients with combined conditions. This is clinically relevant as it not only impacts cardiac function but also the overall prognosis and quality of life in these patients. Additionally, the high prevalence of abnormal electrocardiogram results in the same group further substantiates the critical nature of their cardiac status. These cardiovascular findings are particularly important, given that they correlate with increased symptoms of dyspnea and fatigue, which significantly impair daily functioning and overall well-being.

The presence of more pronounced symptoms such as dyspnea and tiredness in the dual-diagnosis group, as compared with individuals with a single condition, indicates a compounded impact on the quality of life. This not only affects physical capabilities but also likely contributes to psychosocial stressors, impacting mental health and social interactions. The aggregation of these health challenges underscores the need for a holistic approach to treatment and management strategies that address both the respiratory and cardiac dimensions of these patients, especially in the context of recovery from COVID-19. This study’s findings highlight the critical need for integrated care pathways and tailored interventions to improve outcomes for this vulnerable patient population.

Other studies offer detailed insights into the impact of the COVID-19 pandemic on patients with cardiovascular diseases from different methodological approaches. Lim et al. [24] quantitatively analyzed 81 patients with cardiovascular disease, finding that health-related quality of life problems increased from 30% pre-pandemic to 38% during the pandemic. They noted a particular increase in issues related to anxiety and depression, which rose from 12.5% pre-pandemic to 23.5% during the pandemic (*p* = 0.035), with the mean domain-specific score increasing from 1.12 to 1.25 (*p* = 0.012). Radhakrishnan et al. [25], in a qualitative study of 17 older adults with heart failure, highlighted the pandemic’s adverse effects on self-care behaviors such as physical activity. They reported themes of social isolation, financial concerns, and disruptions in access to medications and food, emphasizing how adaptations by the healthcare system and individual health-promoting activities helped maintain resilience.

Truong et al. [26] reported low HRQoL in Vietnamese patients with chronic heart failure, evidenced by particularly low scores in role limitations due to physical health (median 0, IQR 0-25) and general health perceptions (median 25) on the SF-36 scale. In contrast, Caro-Cardon et al. [27] examined post-COVID-19 heart failure outcomes, finding a 27.5% incidence of new heart failure episodes and a 17.6% mortality rate among patients with either clinical heart failure or elevated NT-proBNP. Despite the acute onset and severity of heart issues in the COVID-19 context, quality of life outcomes measured by KCCQ-12 showed no significant differences between these groups, indicating persistent challenges in heart failure management irrespective of the underlying cause or healthcare setting. These studies highlight both the chronic and acute challenges of heart failure, emphasizing the universal impact of the condition on patient quality of life.

In examining the impact of COVID-19 on heart failure management, the studies by Sankaranarayanan et al. [28] in the U.K. and Alharbi et al. [29] in Saudi Arabia highlighted significant disruptions and varying degrees of anxiety among patients. Sankaranarayanan et al. found that 65% of respondents reported disruptions to HF appointments, with 37% experiencing difficulties with medication prescriptions and 34% facing challenges in accessing their HF teams. Anxiety levels were notably higher with respect to COVID-19 compared with HF concerns, particularly among patients under 60 (7.3 ± 2.3 for COVID-19 vs. 6.3 ± 2.2 for HF). Conversely, Alharbi et al., assessed HRQoL among HF patients in Saudi Arabia using the SF-36, revealing that while social functioning scored highly (median 100 points), role-physical functioning was notably low (median 25 points). Education level and gender significantly influenced HRQoL outcomes, with less educated patients more likely to report lower physical component scores, and females more likely to report lower mental component scores. Both studies underscore the profound impact of the pandemic on HF management, though Sankaranarayanan et al. focus more on service disruption and patient anxiety, while Alharbi et al., provide a snapshot of how socio-demographic factors can affect the HRQoL in a non-Western setting.

Rodríguez-Galán et al. [30] and Nishioki et al. [31] presented findings from their respective studies that shed light on the different impacts of COVID-19. Rodríguez-Galán et al. conducted a longitudinal assessment of COVID-19 pneumonia survivors in Spain using the SF-36 questionnaire to measure health-related quality of life. They reported a significant deterioration in HRQoL, with notable declines in the physical role and emotional role dimensions observed 3 and 12 months post-infection compared with the general population. Nishioki et al., on the other hand, analyzed the effect of COVID-19 prevention measures on COPD exacerbations in Japan. They found a notable reduction in exacerbations during the pandemic, with the number decreasing from 36 before the pandemic to 19 during the pandemic. Moreover, they highlighted changes in patient characteristics, noting that the percentage of forced expiratory volume in one second (%FEV1) was significantly higher pre-pandemic at 52.3% compared with 38.6% during the pandemic (*p* = 0.0224), and the body mass index also showed a significant reduction from 22.5 pre-pandemic to 19.3 during the pandemic (*p* = 0.0127). These studies collectively illustrate the pervasive and varied effects of COVID-19 on patients with respiratory conditions, highlighting both the direct impacts on individuals recovering from the virus and indirect benefits from public health measures on chronic respiratory disease management.

Regarding the current study findings, the results should be interpreted in the context of our short-term assessment of only a 3-month follow-up. Long-term follow-up studies of heart failure patients post-COVID-19 reveal substantial ongoing cardiovascular complications. A study noted that a year after recovery, approximately 41.6% of patients continued to experience dyspnea, with some even reporting worsening symptoms, underscoring persistent cardiac issues [32]. Further research highlighted that cardiac MRIs of COVID-19 survivors, conducted months after recovery, showed ongoing myocardial inflammation in 60% of cases and significant heart damage, evident in high levels of blood enzymes like troponin, in 76% of the studied group. These findings highlight the need for ongoing cardiovascular assessment and possibly long-term management strategies to mitigate the extended impacts of COVID-19 on heart health.

Physical rehabilitation post-COVID-19, particularly for patients with chronic conditions, has been shown to significantly improve functional outcomes. A systematic review indicated that various physiotherapy protocols enhanced recovery, with the 6-minute walk test showing marked improvement in functional capacity. For example, in one study, patients who underwent lower-intensity exercises reported significant gains in muscle strength and quality of life measures, with handgrip strength improving notably in the low-intensity aerobic training group (*p* < 0.001) compared with the high-intensity group. Quality of life scores also saw greater enhancement in the low-intensity group (*p* < 0.001) [33].

The findings from this study highlight critical clinical implications for managing long COVID, particularly in patients with coexisting CHF and COPD. These individuals not only face severe HRQoL impairments immediately post-discharge—evidenced by prolonged hospital stays and lower oxygen saturation—but also show significant vulnerability to long COVID symptoms. The observed improvements in HRQoL over three months suggest some recovery but underscore the persistent nature of post-COVID-19 symptoms in this group. This necessitates the integration of long COVID management into care pathways, particularly for those with multiple chronic conditions, to address the complex and ongoing health challenges they face post-recovery.

Based on the current findings, clinicians can help integrate baseline treatment and comprehensive assessments to improve post-COVID-19 outcomes for patients with CHF and COPD. At admission, key clinical parameters such as oxygen saturation (<90%), ejection fraction (<40%), and lung involvement (>50%) should be documented. Follow-up at three months should reassess these parameters and quality-of-life metrics using the EQ-5D and KCCQ questionnaires. Tailored interventions can then be designed; for example, high-risk patients (e.g., those with low oxygen saturation or severe lung involvement) might require intensive rehabilitation and more frequent follow-ups, while low-risk patients might only need standard rehabilitation and routine check-ups. Quantifying improvements, such as a 20-point increase in KCCQ physical limitation scores or a 10% improvement in EQ-5D mobility scores, can help evaluate the effectiveness of interventions. This approach, integrating baseline treatment data and continuous monitoring, aims to enhance long-term outcomes and quality of life for these vulnerable populations.

### 4.2. Limitations

One limitation of this study is its cross-sectional design, which, while effective for assessing the state of HRQoL at a single point in time, does not allow for the observation of changes over time or the establishment of causality among COPD, CHF, their coexistence, and long-term outcomes after COVID-19. This study’s reliance on self-reported questionnaires (EQ-5D and KCCQ) for evaluating HRQoL may also introduce response bias, as patients’ self-assessments can be influenced by their current mood, understanding of the questionnaire, or desire to present themselves in a better or worse light. Additionally, the specific inclusion of patients with CHF and COPD stages NYHA I and II and GOLD A and B may limit the generalizability of the findings to patients with more severe stages of these diseases. Moreover, this study was conducted at a single center, which might limit the applicability of the results to broader populations with varying demographic and healthcare settings. Further studies should also include a control group of patients without CHF or COPD to determine the degree of impact. In addition, the lack of long-term follow-up constitutes a notable limitation. These factors should be considered when interpreting the findings and planning future longitudinal studies to assess the trajectory of HRQoL more comprehensively in this patient population over time. Moreover, the complex treatments involved in these chronic conditions can also have a significant impact on the recovery and symptomatology identified in our study 3 months post-admission, and further studies should assess such implications.

## 5. Conclusions

This study underscores the significant burden of combined chronic heart failure and COPD on health-related quality of life among patients post-COVID-19. The presence of both conditions resulted in notably worse HRQoL outcomes, including longer hospital stays and lower oxygen saturation levels. Importantly, the severity of the initial COVID-19 infection significantly predicted poorer long-term outcomes. These findings highlight the necessity for tailored healthcare interventions that address the complex needs of patients with multiple chronic conditions, aiming to optimize recovery and enhance overall quality of life.

## Figures and Tables

**Table 1:** Background characteristics and demographics.

Variables	CHF and COPD (n = 60)	CHF (n = 60)	COPD (n = 60)	*p*-Value
Age (mean ± SD)	63.81 ± 9.34	65.22 ± 6.25	66.96 ± 7.13	0.221
Female gender n (%)	26 (43.33%)	34 (56.67%)	28 (46.67%)	0.4225
Smoking history n (%)	52 (86.67%)	8 (13.33%)	54 (90.00%)	0.0001
Pack-year smoking (mean ± SD)	22.83 ± 10.55	2.08 ± 2.20	30.86 ± 9.38	0.0003
NYHA categories n (%)				
NYHA I	23 (38.33%)	22 (36.67%)	–	0.7575
NYHA II	37 (61.67%)	38 (63.33%)	–	0.7815
GOLD categories n (%)				
GOLD A	28 (46.67%)	–	36 (60.00%)	0.433
GOLD B	32 (53.33%)	–	24 (40.00%)	0.4495
Comorbidities n (%)				
Hypertension	44 (73.33%)	40 (66.67%)	38 (63.33%)	0.675
Asthma	22 (36.67%)	10 (16.67%)	40 (66.67%)	0.0057
Diabetes mellitus	18 (30.00%)	22 (36.67%)	8 (13.33%)	0.0415
Ischemic heart disease	26 (43.33%)	35 (58.33%)	15 (25.00%)	0.0005
Atrial fibrillation	15 (25.00%)	20 (33.33%)	5 (8.33%)	0.0018
Valve disease	11 (18.33%)	18 (30.00%)	6 (10.00%)	0.0214
Ejection Fraction	55.45 ± 4.68	50.85 ± 6.39	–	0.0006
HFrEF	40 (66.67%)	38 (63.33%)	–	0.781
HFpEF	20 (33.33%)	22 (36.67%)	–	0.757
COVID-19 vaccination n (%)				
1 dose	18 (30.00%)	24 (40.00%)	22 (36.67%)	0.709
2 doses	28 (46.67%)	26 (43.33%)	32 (53.33%)	0.615
3 doses	14 (23.33%)	10 (16.67%)	6 (10.00%)	0.303
COVID-19 severity n (%)				
Mild	18 (30.00%)	34 (56.67%)	26 (43.33%)	0.1255
Moderate	28 (46.67%)	20 (33.33%)	24 (40.00%)	0.513
Severe	14 (23.33%)	6 (10.00%)	10 (16.67%)	0.269
Days of hospitalization for COVID-19 (mean ± SD)	11.63 ± 2.78	8.04 ± 1.61	9.97 ± 2.53	0.0001

SD—standard deviation; CHF—chronic heart failure; COPD—chronic obstructive pulmonary disease; NYHA—New York Heart Association; GOLD—Global Initiative for Chronic Obstructive Lung Disease; HF—heart failure; EF—ejection fraction.

**Table 2:** Disease assessment at admission for COVID-19.

Variables	CHF and COPD (n = 60)	CHF (n = 60)	COPD (n = 60)	*p*-Value
Oxygen saturation (%)	88.7 ± 3.2	92.4 ± 2.1	89.9 ± 2.6	0.0075
Systolic BP (mean ± SD)	151.6 ± 14.8	146.0 ± 13.3	148.7 ± 15.1	0.3215
Diastolic BP (mean ± SD)	87.9 ± 8.3	84.5 ± 7.6	86.1 ± 8.0	0.253
Temperature (mean ± SD)	38.4 ± 0.6	37.9 ± 0.7	38.2 ± 0.5	0.034
HR (mean ± SD)	100.4 ± 12.4	95.6 ± 11.0	98.8 ± 11.6	0.208
Lung involvement on CT (mean ± SD)	56.2 ± 10.6	42.2 ± 15.2	51.5 ± 12.1	0.0002
EF (mean ± SD)	38.4 ± 5.1	40.6 ± 7.4	53.2 ± 6.9	0.0002
Abnormal ECG n (%)	49 (81.67%)	23 (38.33%)	34 (56.67%)	<0.001

SD—standard deviation; CHF—chronic heart failure; COPD—chronic obstructive pulmonary disease; NYHA—New York Heart Association; BP—Blood Pressure; HR—heart rate; EF—ejection fraction; ECG—electrocardiogram.

**Table 3:** Assessment of disease (three months post-COVID-19 admission).

Variables	CHF and COPD (n = 60)	CHF (n = 60)	COPD (n = 60)	*p*-Value
Oxygen saturation (%)	92.6 ± 2.4	94.6 ± 1.8	92.4 ± 2.6	<0.001
Systolic BP (mean ± SD)	144.0 ± 13.2	146.2 ± 10.8	148.2 ± 12.3	0.182
Diastolic BP (mean ± SD)	83.0 ± 6.7	79.5 ± 6.0	81.0 ± 6.5	0.001
Temperature (mean ± SD)	37.3 ± 0.7	36.7 ± 0.6	37.4 ± 0.7	<0.001
HR (mean ± SD)	99.0 ± 11.3	94.3 ± 9.5	97.5 ± 10.2	0.359
Lung involvement on CT (mean ± SD)	22.5 ± 9.0	16.0 ± 7.5	20.5 ± 8.0	<0.001
EF (mean ± SD)	40.6 ± 5.7	42.3 ± 6.9	54.1 ± 5.5	<0.001
Abnormal ECG n(%)	44 (73.33%)	18 (30.00%)	34 (56.67%)	<0.001
Current signs and symptoms n (%)				
Dyspnea	43 (71.67%)	26 (43.3%)	40 (66.67%)	0.003
Edema	35 (58.33%)	30 (50.00%)	20 (33.33%)	0.020
Thoracic pain	24 (40.00%)	10 (16.67%)	16 (26.67%)	0.016
Tiredness	46 (76.67%)	20 (33.33%)	32 (53.33%)	<0.001

SD—standard deviation; CHF—chronic heart failure; COPD—chronic obstructive pulmonary disease; NYHA—New York Heart Association; EF—ejection fraction; ECG—electrocardiogram.

**Table 4:** Analysis of health-related quality of life assessed by EQ-5D.

EQ-5D	Group	At Discharge (Mean ± SD)	At 3 Months (Mean ± SD)	*p*-Value for Time*Group Interaction	*p*-Value for Group Main Effect	*p*-Value for Time Main Effect
Mobility	CHF and COPD	2.87 ± 0.91	2.34 ± 0.82	0.010	0.004	<0.001
	CHF	2.14 ± 0.68	1.67 ± 0.58			
	COPD	2.63 ± 0.78	2.08 ± 0.69			
Self-care	CHF and COPD	2.57 ± 0.97	2.03 ± 0.89	0.033	0.020	<0.001
	CHF	1.92 ± 0.63	1.54 ± 0.52			
	COPD	2.29 ± 0.76	1.87 ± 0.57			
Usual activities	CHF and COPD	3.03 ± 1.02	2.46 ± 0.93	0.008	0.001	<0.001
	CHF	2.18 ± 0.65	1.73 ± 0.59			
	COPD	2.72 ± 0.81	2.19 ± 0.67			
Pain/discomfort	CHF and COPD	2.68 ± 0.74	2.17 ± 0.68	0.027	0.001	<0.001
	CHF	2.07 ± 0.59	1.61 ± 0.47			
	COPD	2.36 ± 0.71	1.94 ± 0.61			
Anxiety/depression	CHF and COPD	2.98 ± 0.94	2.47 ± 0.86	0.009	<0.001	<0.001
	CHF	1.84 ± 0.69	1.38 ± 0.54			
	COPD	2.41 ± 0.73	1.96 ± 0.64			

EQ-5D—EuroQol five dimensions; CHF—chronic heart failure; COPD—chronic obstructive pulmonary disease; SD—standard deviation; BMI—body mass index.

**Table 5:** Analysis of health-related quality of life assessed by KCCQ.

KCCQ	Group	At Discharge (Mean ± SD)	At 3 Months (Mean ± SD)	*p*-Value for Time*Group Interaction	*p*-Value for Group Main Effect	*p*-Value for Time Main Effect
Physical limitation	CHF and COPD	38.94 ± 12.57	58.54 ± 11.93	0.0010	0.006	<0.001
	CHF	53.84 ± 11.29	74.02 ± 9.74			
	COPD	48.38 ± 10.42	68.27 ± 10.64			
Symptoms	CHF and COPD	43.47 ± 14.86	63.73 ± 13.64	0.0026	0.011	<0.001
	CHF	58.91 ± 13.94	78.36 ± 12.26			
	COPD	53.07 ± 12.78	71.52 ± 11.27			
Quality of life	CHF and COPD	41.38 ± 13.64	61.92 ± 12.51	0.0031	0.009	<0.001
	CHF	56.62 ± 11.83	76.38 ± 10.97			
	COPD	50.67 ± 11.53	70.14 ± 9.89			
Social limitation	CHF and COPD	31.97 ± 13.34	51.86 ± 11.68	0.001	0.001	<0.001
	CHF	46.48 ± 12.68	66.69 ± 10.56			
	COPD	42.88 ± 12.04	62.39 ± 10.29			
Self-efficacy	CHF and COPD	46.28 ± 15.92	67.09 ± 14.36	0.001	0.009	<0.001
	CHF	60.96 ± 14.98	81.76 ± 11.67			
	COPD	55.62 ± 13.72	73.24 ± 12.98			

KCCQ—Kansas City Quality of Life Questionnaire; CHF—chronic heart failure; COPD—chronic obstructive pulmonary disease; SD—standard deviation; BMI—body mass index.

**Table 6:** Multivariate regression analysis.

Predictors	Dependent Variable: Usual Activities (EQ-5D)	Dependent Variable: Quality of Life (KCCQ)
CHF	β = −0.147, *p* = 0.325	β = −0.198, *p* = 0.251
COPD	β = −0.097, *p* = 0.408	β = −0.295, *p* = 0.083
CHF + COPD	β = −0.252, *p* = 0.048	β = −0.448, *p* = 0.017
Age	β = −0.019, *p* = 0.492	β = −0.031, *p* = 0.282
Gender (male)	β = 0.177, *p* = 0.239	β = 0.152, *p* = 0.305
Smoking history	β = −0.115, *p* = 0.207	β = −0.163, *p* = 0.165
Hypertension	β = −0.048, *p* = 0.567	β = −0.076, *p* = 0.438
Diabetes	β = −0.088, *p* = 0.373	β = −0.136, *p* = 0.213
Initial severity	β = −0.298, *p* = 0.037	β = −0.342, *p* = 0.024
Length of hospital stay	β = −0.008, *p* = 0.819	β = −0.017, *p* = 0.758

CHF—chronic heart failure; COPD—chronic obstructive pulmonary disease; KCCQ—Kansas City Quality of Life Questionnaire; EQ-5D—EuroQol five dimensions; CHF—chronic heart failure.

## Data Availability

The data are available on request.
